# A Novel Training Program for Police Officers that Improves Interactions with Mentally Ill Individuals and is Cost-Effective

**DOI:** 10.3389/fpsyt.2013.00009

**Published:** 2013-03-18

**Authors:** Yasmeen I. Krameddine, David DeMarco, Robert Hassel, Peter H. Silverstone

**Affiliations:** ^1^Department of Psychiatry, University of AlbertaEdmonton, AB, Canada; ^2^Edmonton Police ServiceEdmonton, AB, Canada

**Keywords:** police, training, mental health, attitudes, behavior, research

## Abstract

Police and law enforcement providers frequently come into contact with individuals who have psychiatric disorders, sometimes with tragic results. Repeated studies suggest that greater understanding of psychiatric conditions by police officers would be beneficial. Here we present a novel approach to training police officers to improve their interactions with those who might have a mental illness. This approach involved developing a carefully scripted role-play training, which involved police officers (*n* = 663) interacting with highly trained actors during six realistic scenarios. The primary goal of the training was to improve empathy, communication skills, and the ability of officers to de-escalate potentially difficult situations. Uniquely, feedback was given to officers after each scenario by several individuals including experienced police officers, a mental health professional, and by the actors involved (with insights such as “this is how you made me feel”). Results showed that there were no changes in attitudes of the police toward the mentally ill comparing data at baseline and at 6 months after the training in those who completed both ratings (*n* = 170). In contrast, there were significant improvements in directly measured behaviors (*n* = 142) as well as in indirect measurements of behavior throughout the police force. Thus, compared to previous years, there was a significant increase in the recognition of mental health issues as a reason for a call (40%), improved efficiency in dealing with mental health issues, and a decrease in weapon or physical interactions with mentally ill individuals. The training cost was $120 per officer but led to significant cost savings (more than $80,000) in the following 6 months. In conclusion, this novel 1-day training course significantly changed behavior of police officers in meaningful ways and also led to cost savings. We propose that this training model could be adopted by other police agencies.

## Introduction

Mental illness is increasing in Canada globally with depression classified as the leading cause of disability worldwide (World Health Organization, 2012). By 2030 the World Health Organization expects that major depressive disorders will be the highest global burden of disease, passing heart disease (World Health Organization, 2008). It is well recognized that police have frequent interactions with mentally ill individuals, but all too often these outcomes can lead to serious injury or death (Patch and Arrigo, 1999; Munetz et al., 2006; Brink et al., 2011). Studies in Canada and the United Kingdom have found that 37–48% of individuals fatally shot by police were classified as having a mental health problem at the time of the shooting (Parent, 1996; Kennedy et al., 1998; Pca, 2003; Best et al., 2004). The fact that there are violent interactions is in part because police officers are increasingly becoming the first emergency responders for individuals with mental illness, and are also assuming magnified responsibilities of both maintenance in the community and referral from the community (Patch and Arrigo, 1999; Lamb et al., 2004). Conversely, individuals with mental illness have very negative attitudes toward the police (Brink et al., 2011) and these may also lead to a more rapid escalation into a violent interaction. Mentally ill individuals are often particularly fearful of the police, and believe that police are likely to use forceful approaches and refuse to listen to them (Watson et al., 2008a). Mentally ill individuals who had poor interactions with the police had increased feelings of hopelessness and a more negative outlook about hospitalization (Jones and Mason, 2002). It is therefore apparent that attitudes of the police, whether real or perceived, can have important impact on subsequent interactions. Lack of understanding and training could also conceivably underlie the high rates of serious injury and fatal outcomes during interactions between police and mentally ill individuals. Training police officers to interact differently therefore may help to solve this issue.

Interestingly, research has also suggested what mentally ill individuals would like to have police trained upon; specifically, “effective communication skills, understanding mental illness and its effects, treating people with compassion and respect, and non-violent conflict resolution skills” (Brink et al., 2011). Adequate police training on mental health issues is becoming increasingly necessary (Psarra et al., 2008). Other studies have re-iterated these findings and believe that police should exhibit the following behaviors: allow individuals a chance to explain themselves; be patient; respond in a calm manner; recognize or ask about mental illness; receive special training to help them respond to people with mental illness more effectively; and keep situations from escalating (Watson et al., 2008a). Many previous studies suggest that training police on verbal intervention strategies and de-escalation techniques would lead to improved outcomes between police and the mentally ill (Keram, 2005; Coleman and Cotton, 2010). Without de-escalation skills, officers may resort to force in order to resolve situations quicker, thus leading to violence and injuries (Ruiz and Miller, 2004). Previous research has also suggested that one component of any successful training program is to have experienced positive personal contact with an individual who has a mental health problem (Penn et al., 1994; Angermeyer and Matschinger, 1996; Corrigan et al., 2001). It is believed that within-group interaction can enhance positive emotions, such as empathy and communication, which can also improve attitudes toward those with mental illness (Brown and Hewstone, 2005; Pettigrew, 2008). However, at present police training has shown limited changes on behavior (Corrigan et al., 2002). The lack of instruments able to assess behavioral change such as verbal communication and de-escalation may contribute to this (Bahora et al., 2008; Compton et al., 2008).

Despite the clear recognition of need, no training program for police officers has met the identified goals. In a recent review of police training programs, which focused on changing attitudes and behaviors (Coleman and Cotton, 2010), it was found that the effectiveness of these training programs was questionable in large part due to the relatively poor quality of the research design testing their effectiveness. It was also noted that there are significant differences in the existing training programs across Canada, the United States, United Kingdom, and Australia.

As an example of differences in current training in Canada, mental health training in 2010 varied widely across the country. Thus it was basic recruit training (Saskatchewan police college), extensive recruit training (Edmonton Police Service, EPS), online courses, hands on practice with mental health professionals, and an officer selective 5-week Police and Crisis Team (PACT) training (Calgary). In Halifax it includes continued education and 40 h of Crisis Intervention Training (CIT). In British Columbia provincial police created an in-depth BC-CIT that includes 17 modules revolving around mental illness awareness, knowledge, and communication, while a less elaborate CIT model is taught to the Vancouver Police. The Ontario Provincial police have basic training, extensive 2 day block training, as well as additional scenario based training with mental illness. The Royal Canadian Mounted Police (RCMP) in certain regions receive suicide prevention training (Nova Scotia, PEI, and New Brunswick RCMP. Other training in Canada can be more comprehensive, for example that of the Peel and Halton Regional Police which includes an extensive 4 days in-classroom training program (originally developed in Hamilton Ontario). Similar differences occur in other jurisdictions, although the primary training in the United States uses either the CIT model (40 h program). In California they use the Police officer Standards and training (POST), an 8-h program consisting of knowledge about mental illness, de-stigmatization strategies, and verbal interventions. England and Wales released a mental health strategy in March of 2010 to improve response to mental illness and criminal justice with mental ill. Emphasis was directed toward the efficient use of police resources, improved information sharing, and partner working. Their training strategy is currently under development. Australia’s training uses using a modified CIT model as well as presentations by person with mental illness. However, validation or research on these training programs is extremely limited.

The training program which has been most widely studied is the CIT model, developed in Memphis, Tennessee (Compton et al., 2008). The essential goal of CIT is described as improving partnerships between police and community mental health resources (Watson et al., 2008b) and “includes education about the causes, signs, symptoms, and treatment of mental illness; substance abuse; psychotropic medication; information on commitment criteria and procedures; consumer rights; personal stories from “consumers” and family members; visits to mental health treatment providers and information about treatment modalities as well as training in communication and de-escalation skills” (Coleman and Cotton, 2010). CIT positive outcomes are described as improved knowledge about mental illness, awareness of community resources (Compton et al., 2006), and improved identification of mental health calls (Teller et al., 2006; Watson et al., 2010). Nonetheless, “there is little outcome research or data-based evidence to inform the exact nature of an effective program, and the research that does exist does not provide guidance as to which components of a learning program are most effective” (Coleman and Cotton, 2010). Furthermore, it was found that majority of all training programs only train specific members, thus not the whole service is getting the knowledge and skills necessary to deal with mental health calls on a daily basis. CIT has been broadly used by police forces in the United States, but it is clear that more research of this program’s potential effectiveness is needed (Compton et al., 2008).

Given this lack of a consistent and well validated training program for police officers that addresses the perceived needs of the mentally ill, and which therefore may help prevent future tragic outcomes, there remains a clear need for a training approach for police officers that is able to change behaviors (Kermode et al., 2009). Therefore, in close collaboration with the EPS, we developed a novel 1-day training program to meet these needs (Silverstone et al., 2013). During this training, pairs of police officers interact with carefully trained actors in one of six well-defined scenarios. These were developed with extensive input from multiple EPS officers to ensure that they were very realistic, but also accurately portrayed mental illness. They were also representative of some of the most common interactions that EPS officers have with mentally ill individuals, and the scenarios had to be designed so that they would not compromise any other police training (for example, on the use of force). Baseline measurements of both attitudes and behaviors was also made, and a variety of linked information was also obtained so that any potential impact of the training could be assessed.

## Materials and Methods

This research was approved by both the University of Alberta Research Ethics Board and the EPS Chiefs Committee. All participating EPS officers gave written informed consent. Baseline measurements were made approximately 4 weeks before any EPS member started the training. Follow-up measurements were made 6 months (±1 month) after the EPS officers had completed their training. All behavioral measurements were also made during the same time periods.

### Training

This is detailed elsewhere (Silverstone et al., 2013), but in brief the 1-day training program was deliberately focused on changing behaviors, and not on increasing knowledge or changing attitudes. This is in contrast to other published training approaches. Thus, there were no theoretical learning sessions or seminars about the conditions that EPS officers would see. Also, before starting each scenario EPS officers received only the typical information they would receive for a call. It should be noted that an extensive amount of time was spent on developing scenarios that could be given in a very similar manner throughout the training period. To develop these scenarios there was extensive interaction between EPS, the Department of Psychiatry, and the actors over 3 months to ensure that the scenarios were effective. The six scenarios that were used in the training program represented the following: a depressed individual who may have taken an overdose; a depressed individual who was very belligerent and potentially violent with a weapon nearby; a psychotic individual who was experiencing hallucinations; an individual with presumed alcohol dependence found collapsing on a public street; an individual with excitement acting strangely on a public street; and a couple who were arguing about the man’s gambling addiction but which also represented other aspects of typical domestic disputes that police officers are called to.

Furthermore, each scenario was not straight-forward, in part to help EPS officers question their preconceptions (for example the individual with presumed alcohol dependence had an alcohol-induced encephalopathy, and it was unclear to the officers if the individual with excitement was in fact showing drug-induced excitation, while in fact they showed typical signs of mania). To help emphasize these issues during the debrief session the EPS officers were asked specific questions about what they observed. The actors were also specifically trained so that the experiences of each pair of EPS officers were as consistent as possible. At every debrief session, discussion revolved around issues of empathy and de-escalation. A unique aspect of the training was that actors specifically informed EPS officers about how their actions had made the actor feel emotionally, e.g., “I was frightened when you came that close to me.” Additionally, for every scenario there were two actors who would portray the mentally ill individual and they would alternate this role. The actor who was not taking part would observe the EPS officers and note their physical movements and empathy. At the debrief session the observing actor would then also give feedback to the specific EPS member about how to potentially improve these interactions. Thus, EPS officers had feedback from two actors, both the one they interacted and with another observing. Throughout the debrief, the primary focus of feedback was on helping the EPS member be more empathic as well as helping them identify other approaches they may have used to de-escalate the situation. All feedback was collected on an on-going basis in a standardized manner to ensure that each scenario and debrief were as consistent as possible.

It should be also noted that, prior to this training, EPS officers had only received specific training on mental health issues before they graduated as a police officer. There had been no previous specific mental health training once an officer had graduated.

The cost of developing the training program was not captured, but involved significant time from many members of the EPS training unit, particularly, as well as the University of Alberta staff involved in the study. However, the costs of running the training program were captured. The largest component was having two actors present for every scenario on all 19 training days. The total external costs (not including the cost for EPS training staff or University of Alberta Staff) was $70,000 plus other costs of approximately $10,000. As there were 663 EPS officers who received the training the average cost was therefore $120. However, this would fall with future programs for efficiency reasons as not every session was fully booked. Secondly, it would be possible to decrease these costs by only having one actor present for each scenario. This would likely mean that future training programs will likely average between $60 and $100 per officer (not including the time for EPS training staff).

### Measurement of attitude

Two scales were used to determine attitudes of EPS officers to mentally ill individuals.

#### Community attitudes toward mental illness

The Community Attitudes toward Mental Illness (CAMI) scale was designed to measure the degree of social stigma toward mental illness (Taylor and Dear, 1981). It has been widely used in research in different populations (Morris et al., 2012). The CAMI consists of four subscales, two of which measure stigma [called “Authoritarianism” (CAMI-A) and “Benevolence” (CAMI-B)], one measuring fear of the mentally ill [called “Social Restrictiveness, SR” (CAMI-SR)], and one measuring acceptance of the mentally ill in the community [called “Community Mental Health Ideology, CMHI” (CAMI-CMHI)]. Each subscale contains 10 statements and are answered using a five-point Likert scale ranging from “strongly disagree” (1) to “strongly agree” (5).

#### Social distance scale

The Social distance Scale (SDS) measures the level of social distance, or the desired degree of social distance, between a member of one social group and the members of another (Bogardus, 1933). In the present study we used a modified version (Link et al., 1987) with three specific vignettes (Jorm et al., 2005) applicable to the scenarios being studied. These vignettes described individuals who had substance abuse, depression, or schizophrenia. After reading each vignette, there are five statements regarding the reader’s willingness to interact with this individual (such as would you “Like to move next door to a person like this,” or “Recommend a person like this for a job?”). Measurements are determined using a five-point Likert scale ranging from “definitely willing” (1) to “definitely unwilling” (5). Higher scores indicate more social distance and lower scores indicate less social distance (range 1–5).

#### Measurement of knowledge

Two scales were used to determine EPS officer knowledge about mental illness.

#### Mental illness recognition scale

A list of 27 ailments were listed and officers were asked to determine which of these they believed to be a mental illness (i.e., Those who have a “nervous breakdown,” “schizophrenia,” “Autism” etc). Of these 27, only 9 are considered mental illnesses according to DSM-IV. Measurements were determined by subtracting the number of right answers from the number of wrong answers, with the possible score range being from −18 to +9 (as they could leave answers blank).

#### Mental illness knowledge

A total of 16 multiple-choice questions were used to measure police officers detailed mental health knowledge. Questions such as “The term ‘Clinical Depression’ refers to,” or “Which of the following illnesses usually involves a psychotic episode?” were used, with scores ranging from 0 to 16.

### Behavioral measures

#### Supervising officer survey

For every EPS officer who took part in the study we asked their direct supervising officer (normally a sergeant) to rate the individual officer at baseline on a number of behaviors that they observed. This was done on an anonymous basis, with each officer only having a study number reported to the study team (so we could compare to subsequent data). They were specifically asked to rate the ability of the EPS officer to communicate with the public, their ability to verbally de-escalate a situation, and their level of empathy in dealing with the public. Measurements were determined using a five-point Likert scale ranging from “negative and needs improvement on more than one occasion” (1) to “extremely positive in most situations, and is better than the average officer” (5). Higher scores indicate better ability of officers to communicate and interact with individuals (range 1–5). An increase in the ability of officers to improve communication skills, ability to de-escalate situations, and to increase empathy during interactions were primary outcome goals of the training program.

#### Number of mental health calls

Another method to measure change in behavior was to determine how many calls EPS officers identified as being due to mental health issues, since awareness of the potential of an individual having a mental illness was a secondary outcome goal of the program. An increase in the number of calls being identified as being due to mental health issues would therefore suggest a positive outcome of the training program.

#### Time spent on mental health calls

The amount of time that each EPS officer spends on every activity is documented (in minutes). It was anticipated that if EPS officers become more confident in their interactions with mentally ill individuals, that they may become more efficient in the use of their time when dealing with mentally ill individuals. A secondary outcome goal of the training program was therefore to achieve a reduction in the time taken per mental health call.

#### Use of force

There are a wide range of measurements of the use of force. The first involves the use of weapons, and the following events fall into this category: firearm, fired; impact, specialty munition; conduction energy weapon (CEW; proverbially known as a “taser”), CEW Stun; Impact, Other; Physical, Disarming Technique; CEW, CEW Probes; Canine Presence; Canine, Canine Contact; OC, OC Deployed; Impact, Baton Deployed; Firearm, Pointed; Firearm, Low Ready; CEW, CEW Presence/Laser. The second category are the use of physical force and this is classified as one of the following events: physical, strike; physical, stun; technique/distraction; physical, joint manipulation; physical, balance displacement/takedown; physical, holding technique. One of the goals of the training program was to decrease the use of both weapons and physical force in all calls involving the mentally ill. The total for all types of force was used in the calculations, which determined how frequently any kind of force was used in an interaction with a individual (as a percentage of the total number of mental health interactions).

### Other measures

#### Changes in spending on EPS officer interactions with the mentally ill

Knowing the cost per hour of each officer it was possible to calculate any cost-changes following the program. If the training program ended up leading to more efficient interactions between EPS officers and mentally ill individuals, such that EPS spent less time per call, this would translate into cost savings. In contrast, if the training program didn’t have this benefit, but instead increased the amount of time spent with mentally ill individuals then the training program would have additional costs for the EPS.

#### Anecdotal community feedback

In addition to the formal measures, we carried out some enquires to determine anecdotal feedback. Thus, approximately 2 months after the training was completed we asked some of the key individuals in the community who work with homeless mentally ill in Edmonton some non-directive questions about how they found the police interactions with the population they dealt with. We didn’t explain that we had completed a training program, only that we were interested in their views.

#### Statistical analysis

For comparisons between baseline measurements of attitudes and those from the same individuals 6-months later we used paired *t*-tests for the CAMI and mental illness knowledge while the Wilcoxon Signed Rank Test was used for the SDS.

To compare changes we used summaries of the monthly data for the 6 months after the training was completed (July–December, 2011) and compared these to the same 6 months periods in 2010 and 2009 (the only years for which similar data was available). This was used to determine changes in the time spent per call, recognition of mental illness and officer supervisor surveys (Wilcoxon Signed Rank Test) as well as for the number of mental health calls and changes in the use of force (paired *t*-tests).

For all statistical analysis we used SPSS Version 19 (SPSS Inc., Chicago, IL, USA).

## Results

A total of 663 police officers participated in the mental health training, of whom 312 completed baseline assessments and 372 completed assessments 6-months post-training. Of these, 170 EPS officers completed assessments at both baseline and 6 months and therefore changes in individual ratings could be made for these 170 police officers, who form the study sample for the comparison data on attitudes.

The baseline sample (*n* = 312) had an average age of 31.4 years (SD = 6.3), with 87.6% being male and 12.4% female. Most of the police in the training program were not very experienced. Thus, the length of time they had been a police officer ranged was 0–2 years (55%), 3–5 years (36%), or more than 5 years (9%).

In terms of education, 30.2% had completed high school, 57.4% had received a post secondary degree or certificate, and 12.4% had earned a graduate degree. The sub-set of 170 officers who completed both samples did not significantly differ from the larger sample on any measurement.

Overall, the training was well received (Silverstone et al., 2013). At baseline, police officers were asked if they believed they had sufficient training about mental illness. At baseline 44% of officers believed they had sufficient training in dealing with mental illness. However, at 6-months post-training this belief was increased to 67%, a 23% increase (*p* < 0.001).

We found no statistically significant differences on any measure between the 170 who completed both assessments and those who completed only the baseline or follow-up assessments. We therefore believe that the 170 individuals are representative of the larger sample.

### Community attitudes toward mental illness

**Figure 1:**
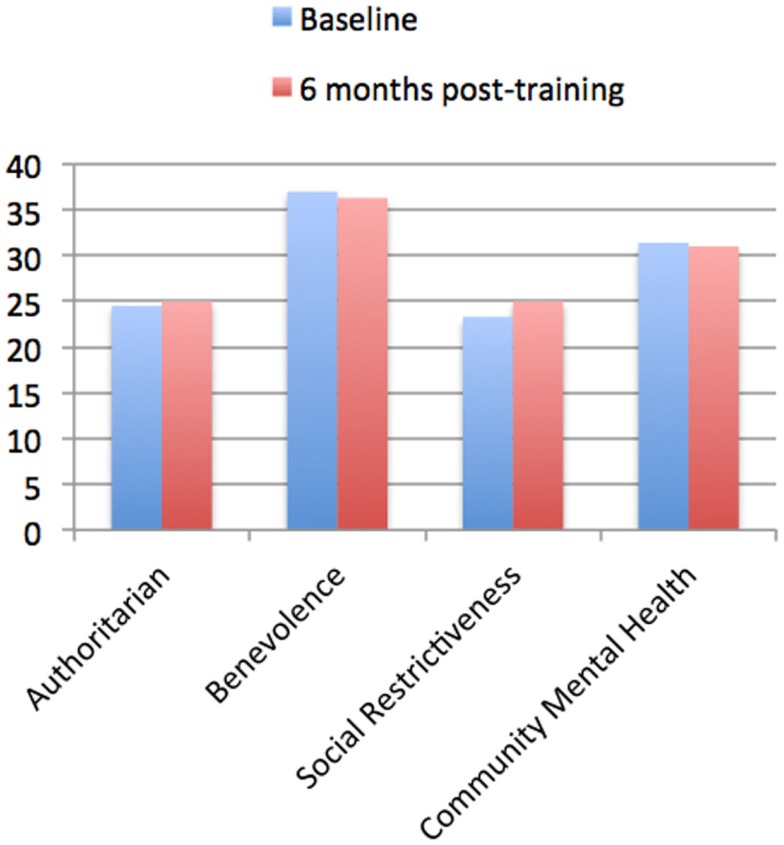
**Community Attitudes toward Mental Illness (CAMI) questionnaire at Baseline vs. 6 months post-training**. The results for the CAMI questionnaire are shown for police officers who completed both baseline and 6-month follow-up ratings (*n* = 170). Four subscales of the CAMI were used which measure attitudes toward the mentally ill; the “Authoritarian” subscale measures pessimistic attitudes, the “Benevolence” subscale measures compassion and empathy, the “Social Restrictiveness” subscale measures the level individuals feel that people with mental illness are dangerous and threatening, while the “Community Mental Health Ideology” subscale determines the level of acceptance toward deinstitutionalization and attitudes toward having mental health facilities in the community. Higher scores on “Authoritarian” and “Social Restrictiveness” signify greater amounts of stigma, while higher scores on “Benevolence” signifies lower stigma and a higher score on “Community Mental Health Ideology” signifies higher acceptance of the mentally ill. Data are shown at “Baseline,” i.e., before police officers were trained, and these are compared to “6 months post-training.” Community samples reported in the literature, defined as “controls” from Finkelstein et al. (2008) are 25 (CAMI-A), 40.9 (CAMI-B), and 26.3 (CAMI-SR) respectively. No control data is available for CAMI-CMHI. It can be noted that the police officers ratings appear similar (and slightly lower) than those of the community Controls, and there appear to be possibly slightly lower stigma in police officers compared to community Controls.

At baseline the combined average scores of the 170 police officers for the four subscales of the CAMI were 24.5 ± 4.9 (CAMI-A), 37 ± 5.1 (CAMI-B), 23.3 ± 5.1 (CAMI-SR), and 31.4 ± 5.6 (CAMI-CMHI). There were no statistically significant differences from baseline at 6 months post-training (Figure 1), and scores for the four subscales of the CAMI were 25 ± 4.7 (CAMI-A; *p* = 0.156), 36.3 ± 5.9 (CAMI-B; *p* = 0.107), 25 ± 5.4 (CAMI-SR; *p* = 0.130), and 31 ± 6.1 (CAMI-CMHI; *p* = 0.294).

### Social distance scale

**Figure 2:**
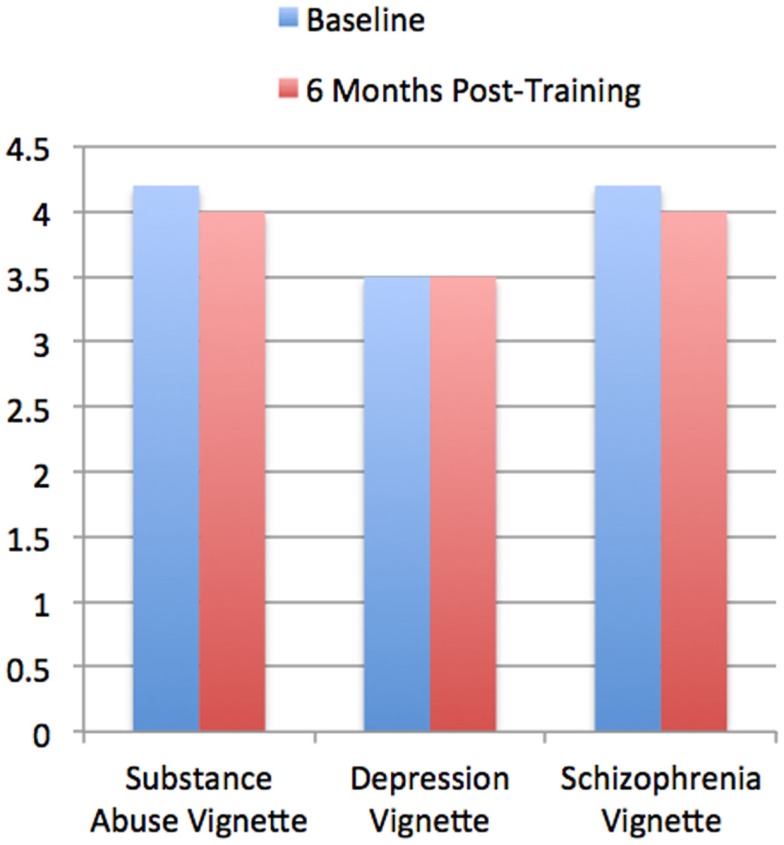
**Social Distance Scale (SDS) at Baseline vs. 6 months post-training**. The version of the SDS used in the present study contained three scripted vignettes about schizophrenia, depression, and substance abuse followed by statements such as “Would you be willing to move next door to John?” The data is from police officers who completed both baseline and 6-month follow-up ratings (*n* = 170). Baseline measurements were determined using a five-point Likert scale ranging from “definitely willing” (1) to “definitely unwilling” (5). Higher scores indicate more social distance and lower scores indicate less social distance (range 1–5). Data are shown at “Baseline,” i.e., before police officers were trained, and 6 months post-training. Community samples reported in the literature, defined as “controls” from Link et al. (1999), Nordt et al. (2006) are 3.2 (substance abuse), 2.5 (depression), 3 (schizophrenia). It can be seen that the police officers ratings at Baseline appear higher to those of the community Controls, i.e., that there appears to be greater stigma among the police officers compared to community Controls.

At baseline the combined average scores of the 170 police officers for SDS baseline measurements were 4.2 ± 0.8 for the drug abuse vignette, 3.5 ± 0.9 for the depression vignette, and 4.2 ± 0.9 for the schizophrenia vignette. There were no statistically significant differences from baseline at 6 months post-training (Figure 2) with post-training SDS scores being 4.0 ± 1.0 for the drug abuse vignette (*p* = 0.230), 3.5 ± 1.0 for the depression vignette (*p* = 0.395), and 4.0 ± 1.0 for the schizophrenia vignette (*p* = 0.180).

### Mental illness recognition scale

At baseline the mean recognition scores of 170 police officers were 1.9 ± 2.8 with post-training recognition scores being significantly lower at 1.3 ± 2.9 (*p* = 0.011). However, since this is an unvalidated measure it is uncertain whether or not this signifies a meaningful increase in knowledge.

### Mental illness knowledge

At baseline the mean knowledge scores of 170 police officers were 8.4 ± 2.6 with post-training knowledge scores being 8.7 ± 2.7 (*p* = 0.127).

### Supervising officer survey

**Figure 3:**
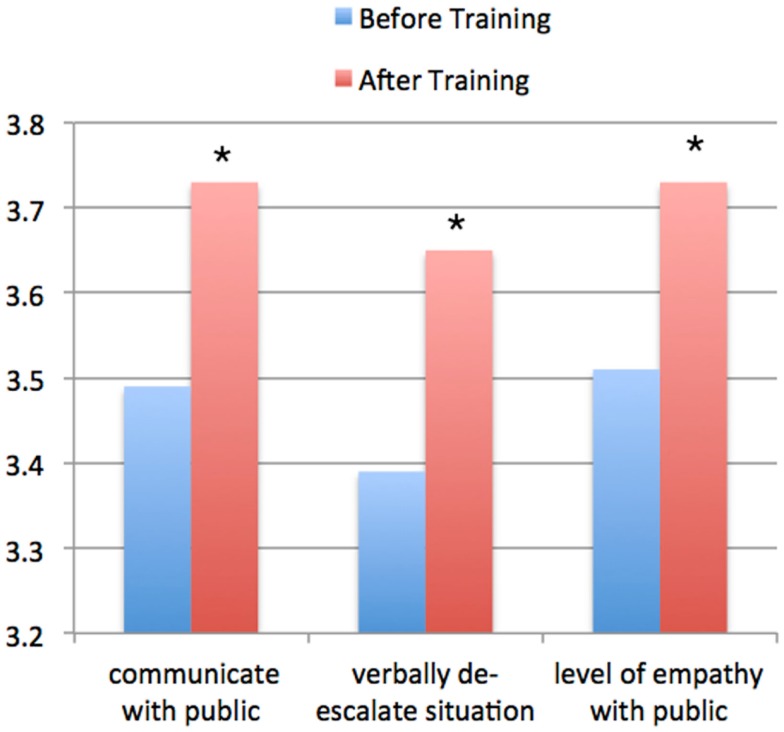
**This shows the data of observed behavior by the supervising officers of individuals (*n* = 142) who provided feedback on individual officer ratings on the following three measures when interacting with individuals who potentially had a mental illness: the ability to communicate with the public, the ability to verbally de-escalate a situation, and their demonstrated level of empathy when interacting with the general public**. Data are shown at “Baseline,” i.e., before police officers were trained, and 6 months post-training. There were statistically significant differences on all three ratings (*p < 0.005).

A total of 142 officers had ratings by their supervising officer at both baseline and at the 6-month follow-up, and these are the sample for measured changes in behavior. Comparing these 142 to the total sample of 312 who completed baseline measurements. These showed highly statistically significant increases which reflected an approximately 10% improvement in ratings (Figure 3). In terms of rated ability of individual police officers to communicate well with the public there was an improvement from a baseline rating of 3.49 ± 0.86 to a 6-month rating of 3.73 ± 0.77 (*p* = 0.001). In terms of the police officer’s ability to verbally de-escalate a situation there was an improvement from 3.39 ± 0.87 to 3.65 ± 0.79 (*p* = < 0.001). In terms of the police officer’s level of empathy in dealing with the public there was an improvement from 3.51 ± 0.73 to 3.73 ± 0.73 (*p* = 0.003).

It should be noted that in terms of these changes, a 10% improvement would be considered meaningful given that that the range is from 0 to 5, and so there is the potential problem of a “ceiling effect” with the baseline being at 3.5 (a ceiling effect occurs when scores are at or near the maximum possible score for the variable being measured).

### Indirect and other measures

It should be noted that this data is from information across all of the EPS. It includes feedback both from those who attended training (*n* = 663) and those who did not. However, since over 90% of “beat officers” attended training, and these officers formed the vast majority of individuals responding to these calls, we believe that the following indirect measures represent changes that were due to training.

Additionally, all indirect behavioral measures compare the same 6-month period (July–December) for the years 2009–2011. This is because the training took place throughout the second quarter of 2011 and thus it isn’t possible to accurately compare the first 6-months of these 3 years. Additionally, the questionnaire data and the supervising officers survey also relates to this time period. For this reason we have only compared data for the 6 months period following training to the same period in the two previous years (the only ones for which similar data was available).

### Number of mental health calls

**Figure 4:**
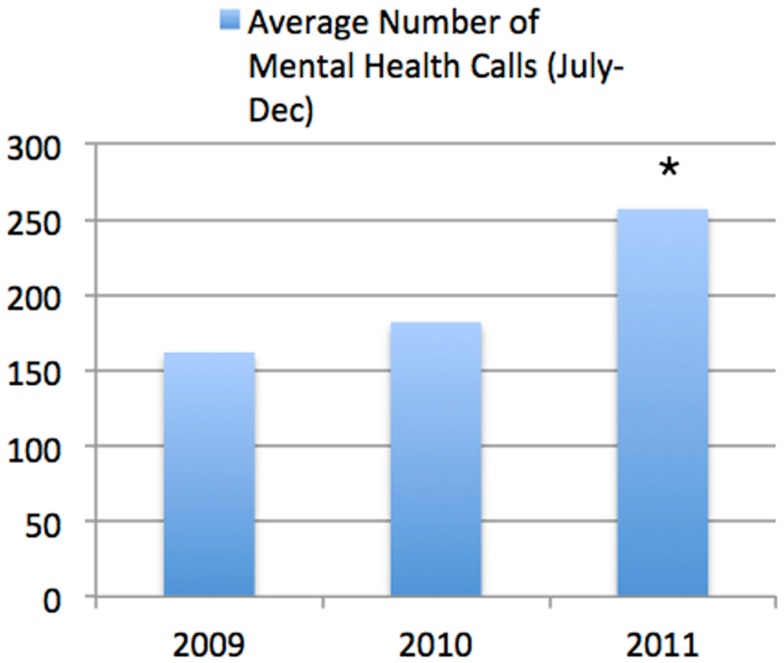
**Average Number of Mental Health Calls (July–December)**. This shows the information across all of the Edmonton Police Force. It can be seen that there was a highly significant increase in the number of calls identified as being due to mental health issues between the same time period in 2010 compared to 2011 (*p < 0.001).

The average number of mental health calls during the period from July to December each year increased from 2009 (*n* = 162) to 2010 (*n* = 182) and onto 2011 (*n* = 257; Figure 4). It was statistically significant when comparing both changes from 2009 to 2010 (*p* = 0.031) and 2010 to 2011 (*p* < 0.001).

### Time per mental health call

**Figure 5:**
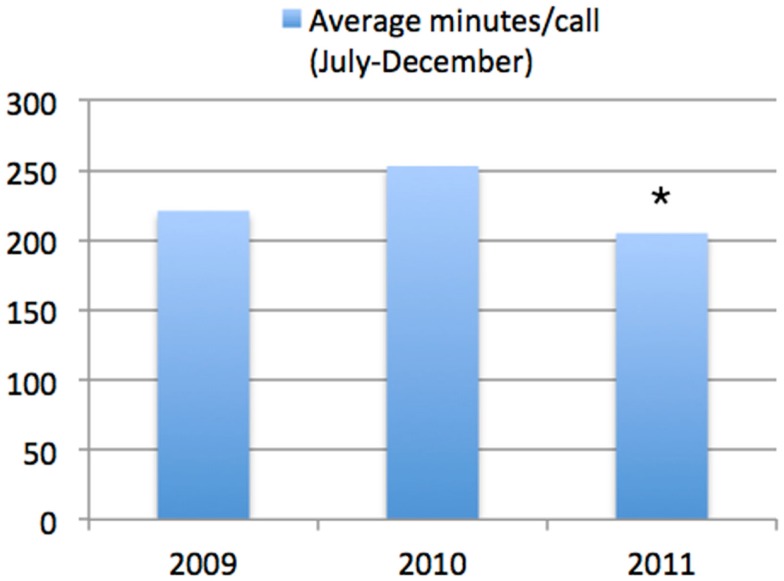
**Average minutes per call (July–December)**. This shows the information across all of the Edmonton Police Force. It can be seen that there was a highly significant decrease in the time taken per mental health call between the same time period in 2010 compared to 2011 (*p < 0.001).

The average time taken for each mental health calls during the period from July to December each year increased significantly from 2009 (221 ± 142 min) to 2010 (251 ± 164 min; *p* = < 0.001; Figure 5). In marked contrast, there was a statistically significant decrease from 2010 (251 ± 164 min) to the same period in 2011 (205 ± 146 min; *p* < 0.001).

### Number vs. time

**Figure 6:**
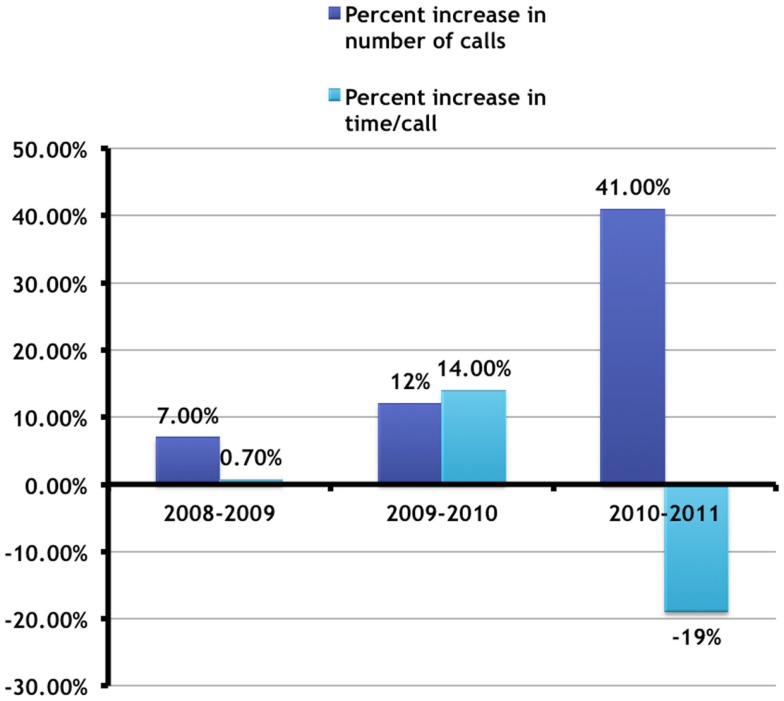
**Change in number vs. time per call (July–December)**. This combines the data from Figures 4 and 5 and shows these changes as a percentage change from the previous years during the same time period. It can be seen that there were marked changes from 2010 to 2011 in that while the number of calls increased significantly, the time taken per call decreased significantly. This did not happen in previous years, and suggests an increased identification and increased efficiency of police officers in interacting with the mentally ill during the 6 months following training.

Combining the data from Figures 4 and 5 demonstrates a marked change in behavior compared to previous years (Figure 6).

### Use of force

**Figure 7:**
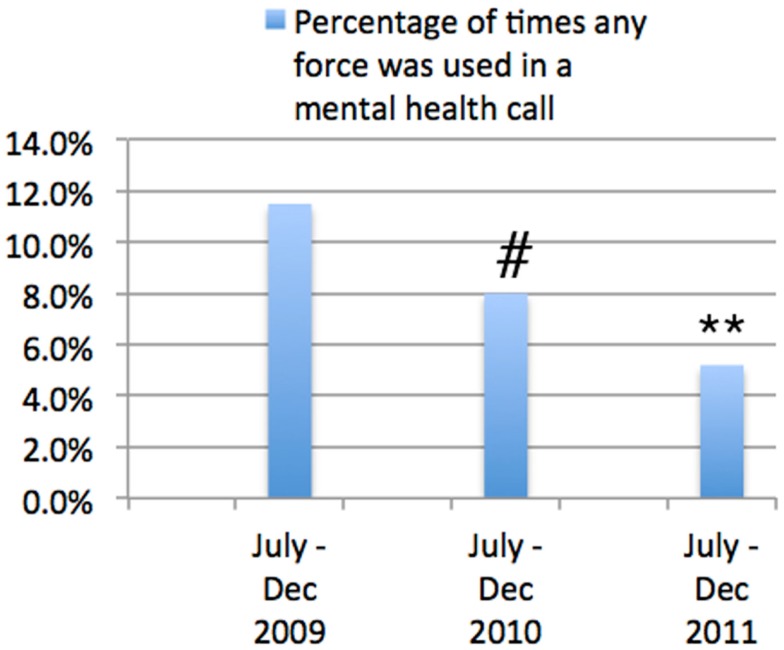
**Change in the Use of Force in mental health calls (July–December)**. This shows the percentage of times that any type of force was used in a mental health call. It can be seen that there was a highly statistically significant decrease in the percentage of times force was used both between 2009 and 2010 (#*p* < 0.001) and between 2010 and 2011 (**p < 0.001). However, it should be noted that there were other training activities in the 6-month period (September 2010–March 2011) prior to the current training program that are also likely to have impacted this reduction in the use of force. It is therefore likely that the current training program was one contributor to this significant reduction, but not likely to be the sole reason for this reduction.

When comparing the same time periods from 2009 to 2011 there was a striking decrease in the use of force for individuals who had a mental illness (Figure 7). Thus, the percentage of times force was used in any Mental Health Call decreased from 11.5 ± 1.9% in the July–December period of 2009 to 8.0 ± 1.2% in the July–December period of 2010 (*p* = 0.011), with a further reduction to 5.2 ± 0.9% in the July–December period of 2011 (*p* = 0.004).

However, it needs to be recognized that on this specific issue there were a number of other initiatives introduced by the Edmonton Police Force during the period September 2010–March 2011 to specifically reduce the use of force in all circumstances, and it is thus highly unlikely that the significant reduction in use of force was due solely to the training program, which should be seen as one element of this successful approach.

### Cost-effectiveness of training program

The training program had two major costs, internal and external to the EPS. The internal costs were those for the training and time by the members of the EPS. The primary resource was use of time by the training division. Given that this group was already committed to provide training during the same period, it is assumed that there was no additional training cost time. However, this is unlikely, since learning a new training routine always takes time, but this additional cost has not been factored into the overall budget. Another “internal” cost was the cost for the researchers and the time they spent developing the program and training the actors. Again, however, these costs were supported by other means and so have not been considered in the total cost profile.

The other costs were external costs. The primary one was the cost for actors to be trained and to be in attendance. The rates used were standard rates for actors and published by the appropriate representative organizations. In this particular training scenario we chose to have two actors available for every role, for a total of 14 actors each day (five scenarios had one actor and one scenario used two actors so each day there were seven actors who would carry out a scenario with seven others watching. They would rotate after each set of scenarios to allow the scenario to remain consistent by avoiding “burn-out” in the actors). Thus, one actor (or pair of actors) carried out the training while the other watched and gave comments back to the EPS officers. It is unclear if this additional cost was necessary, as we did not carry out training without this occurring. However, using this approach, to train a total of 663 officers on 19 separate training days, and with several days of advance training of the actors, the cost was slightly less than $80,000 CDN (including all miscellaneous costs which totaled less than $10,000). Thus, the cost per officer was approximately $120. If fewer actors were used the cost would be decreased proportionately. In terms of cost-effectiveness one way of measuring this would be to determine the difference in costs if the training had not led to an increased efficiency (i.e., the amount of time spent per mental health call decreased), which is in marked contrast to previous years. Thus, if the time spent per mental health call in 2011 was the same as the time spent in 2010, then there would have been additional expenditure of approximately $84,000 in the 6-month period from July to December 2011. This suggests that the training is cost-effective.

## Discussion

The goal of this training program was to improve interactions between police and mentally ill individuals. The focus of this novel training scheme differed from previous training programs in that it was focused on a few behavioral changes: to increase the abilities of police officers to appear empathic; to communicate better with those who have mental illness; and to be able to better de-escalate potentially violent situations. This has not been studied well previously, despite its obvious importance (Angermeyer and Dietrich, 2006). Interestingly, the results suggest that the training program was successful in meeting these goals, even though there was no underlying change in the attitudes of the police officers toward those with mental illness. Thus, we were able to show a highly statistically significant improvement in police officer behavior some 6-months after a 1-day training program. This improvement was reflected in both direct measurements and indirect assessments, and led to significant cost savings that make the training program clearly cost-effective. Interestingly, it was previously described that it is currently unknown whether a decrease in attitudinal stigma relates to the actual behavior of police officers toward those with mental illness (Jorm and Griffiths, 2008), and our results suggest that changing stigma or understanding of mental illness is not necessary to change behavior.

Importantly, this training led to measurable improvements in officer interactions with individuals who had mental illness. It is quite possible that there were also improvements in behavior with other members of the public, although this was not measured and must therefore remain speculative. However, one of the issues that arises from this training is how could a single day’s training, which didn’t impact attitudes at all, have a consistent and measurable impact on behavior in stressful situations at least 6 months later? We believe that this is due to the unique and novel nature of the training. Specifically, this training program created scenarios in which officers were emotionally engaged as well as intellectually engaged, and despite the somewhat artificial nature of the interactions the ability of the high quality actors to engage the officers we believe is the largest single reason why this training program was so successful. An anonymous comment about the training program that reflects the potential benefits supports this hypothesis, in which a supervising officer stated: “Great day, of a particular note, a member of my squad mentioned several days later that they had gone to a call “exactly like one of the scenarios” as such, they were able to reference what they learned in achieving a successful outcome.” In terms of the impact of the training other anonymous comments give some of the qualitative impact of the training. Thus:

“This was an excellent training day that provided as close to the real world situations as possible while maintaining a safe environment. The actors were extremely professional and their input and feedback was very beneficial for the learning objectives.”

“I felt that this was an excellent training day. It kept everyone engaged throughout and the scenarios were excellent. Having a variety of scenarios was great for the members.”

“I like the idea of feedback and doing scenarios to assist with our abilities on the street. It was great to have both an inside police perspective and a civilian perspective of how we conduct our investigations.”

“By far the finest training day I’ve ever attended. This type of format allows supervisors an accurate assessment of how their members react on the street. I believe this training endeavor will give our members more confidence and therefore allow them a higher-level proficiency when making decisions based on risk management.”

This degree of active engagement may, in part, suggest why such a brief period of training could have such a long-term positive outcome. There is an extensive body of research demonstrating that role-play and simulation can be very effective tools in teaching psychiatry (Barney and Shea, 2007; McNaughton et al., 2008), and others have specifically advocated experiential training through actors in role-play situations (Ballon et al., 2007), although the effectiveness of these approaches have not been formally tested over a prolonged period. Similarly, others have shown that it is possible to improve empathy using actors who are role-playing being patients, and that these improvements last at least 1 month (Walters et al., 2007). Additionally, it has long been clear that emotion may enhance memory processes that occur at all stages, including encoding, storage, and retrieval (Labar and Cabeza, 2006; Okada et al., 2011). The scenario’s were specifically designed to increase emotional arousal of the police officers, and this is also likely to have increased the learning that took place from six consecutive scenarios all of which emphasized the same points (empathy, communication, de-escalation of potentially difficult, or violent behavior). One of the other unique aspects of this training may also have helped in the beneficial outcomes, and this was that in most cases supervising officers took part in the training as well. Thus, they learned the key aspects of what the training program expected from the police officers they were supervising, and we believe (from informal feedback) that this may have helped ensure that the learning was continued. While this is currently speculative at this time, it is worth determining in future research whether or not the involvement of the supervisors contributes at all to the positive outcomes seen.

Thus, in conclusion, we believe that the present findings build on previous studies, and show that a specific and targeted role-playing training session for police officers can meaningfully improve interactions between police officers and the mentally ill over the longer-term. This leads to a measurable change in behavior, and may possibly also decrease the use of force in interactions between police officers and those with a mental illness. Given the cost per officer of $120, and the findings that these costs are likely to be saved in a short period of time, we believe that the present research strongly supports the use of this training program, in other police forces.

Limitations of this study include the facts that we utilized anonymous self-report measures in attitudes, and supervisor surveys, and we did not carry out interviews. Furthermore, we did not get specific feedback from individuals with whom the police officers interacted, so were not able to measure possible changes in perception of these individuals. This aspect, which has scarcely been studied previously, will need to be addressed in future research.

## Conflict of Interest Statement

This research was supported in part by a grant from the Edmonton Police Services.
